# Chest Wall Perforator Flaps in Breast Conservation: Versatile, Affordable, and Scalable: Insights from the Largest Single-Surgeon Audit from India

**DOI:** 10.3390/curroncol32030165

**Published:** 2025-03-14

**Authors:** C. B. Koppiker, Rupa Mishra, Vaibhav Jain, Priya Sivadasan, Chetan Deshmukh, Beenu Varghese, Upendra Dhar, Anushree Vartak, Namrata Athavale, Neerja Gupta, Laleh Busheri, Vishesha Lulla, Sneha Bhandari, Sneha Joshi

**Affiliations:** 1 Prashanti Cancer Care Mission (PCCM), Pune 411016, India; rupamishra@prashanticancercare.org (R.M.); cda1@prashanticancercare.org (V.J.); priyasivadasan@prashanticancercare.org (P.S.); namrata.athavale@prashanticancercare.org (N.A.); laleh.busheri@prashanticancercare.org (L.B.); oncopsychologist@prashanticancercare.org (V.L.); cdc4@prashanticancercare.org (S.B.); 2 Centre for Translational Cancer Research, Joint Initiative by Indian Institute of Science Education and Research (IISER) Pune and Prashanti Cancer Care Mission, Pune 411008, India; 3 Jehangir Hospital, Pune 411001, India; 4 Orchids Breast Health Centre, in Association with PCCM and Jehangir Hospital, Pune 411001, India; chemotherapy@prashanticancercare.org (C.D.); radiology1@prashantcancercare.org (B.V.); upendradhar@hotmail.com (U.D.); 5 International School of Oncoplastic Surgery, Pune 411048, India; oncoplastyfellow2@prashanticancercare.org (A.V.); isosfellow@prashanticancercare.org (N.G.)

**Keywords:** perforator flap, oncoplasty, breast cancer, quality of life, LMIC

## Abstract

Chest wall perforator flaps (CWPFs) are a promising option for partial breast reconstruction but are underutilized, particularly in resource-limited settings. This retrospective observational study explores the feasibility and impact of CWPFs in breast-conserving surgery at our single-surgeon center, where 203 procedures were performed between 2018 and 2023. We evaluate 200 cases treated after multidisciplinary tumor board discussions and shared decision-making, assessing clinicopathological data, surgical outcomes, oncological results, cosmetic outcomes, and patient-reported outcome measures (PROMs). The median age of patients was 52.5 years. Single CWPFs were used in 75.9% and dual flaps in 24.1%. Sentinel node biopsy was performed in 76.9% of malignant cases, with no positive margins. Minor complications occurred in 11%, and no major complications were reported. At a 27-month median follow-up, the overall survival rate was 97.5%, with a disease-free survival of 92.1%. Cosmetic outcomes were good-to-excellent, and PROMs indicated high satisfaction. This largest single-surgeon study from Asia demonstrates the transformative role of CWPFs in breast conservation surgery for Indian women with sizable, locally advanced tumors. The technique offers excellent oncological and cosmetic outcomes, reduced costs, and a shorter operative time, highlighting the need for oncoplastic algorithms in resource-limited settings to improve breast conservation accessibility.

## 1. Introduction

Breast cancer is a leading cause of cancer-related morbidity and mortality globally, with a pronounced burden in developing countries such as India. Over the past few decades, the incidence of breast cancer has been steadily increasing, and it has now surpassed cervical cancer as the most common malignancy among Indian women. This epidemiological shift poses a significant challenge for the healthcare system, compounded by the fact that breast cancer in India often presents at advanced stages and in younger women compared to Western populations [1,2,3,4]. These factors necessitate the exploration and implementation of innovative treatment strategies that address not only the disease itself but also the unique sociocultural and economic challenges associated with breast cancer care in resource-limited settings.

Breast conservation, or lumpectomy, focuses on removing tumors while meticulously preserving healthy breast tissue, maintaining natural contours and aesthetics. The primary focus of our center is breast tissue preservation, with breast-conserving surgery (BCS) performed in approximately 80% of cases. This approach, whether standalone or supported by oncoplastic techniques, prioritizes oncological safety while ensuring superior cosmetic outcomes. Large cohort studies have shown that this approach offers psychological benefits, better body image, and comparable survival rates to mastectomy when coupled with adjuvant therapies such as radiotherapy, for patients with tumors under 4 cm [5,6,7,8].

However, advanced-stage tumors or significant tissue removal can complicate BCS, often leading to contour deformities and dissatisfaction, making mastectomy the common recommendation in such cases, a regular reality in the Indian context [9]. In such cases, oncoplastic surgery has revolutionized breast conservation by combining oncologic principles with reconstructive techniques to optimize both cancer control and aesthetic outcomes. Through volume displacement and replacement strategies, oncoplasty allows tissue reorganization or autologous tissue addition to fill defects post-tumor excision. These techniques have expanded BCS indications, enabling satisfactory results even for larger tumors or smaller breasts. Among these, perforator flaps have shown great promise in partial breast reconstruction [10,11,12,13,14].

Perforator flaps are tissue segments that maintain their blood supply through perforating vessels, enabling the harvesting of skin and fat without muscle, minimizing donor site morbidity while providing adequate volume for reconstruction. Initially used in free flap reconstructions post-mastectomy, perforator flaps are now increasingly applied in partial breast reconstructions as pedicled flaps. Chest wall perforator flaps (CWPFs), a subset of perforator flaps, are particularly effective for partial breast reconstruction in patients with small-to-moderate-sized breasts. Although their use in breast cancer surgery is relatively recent, emerging evidence highlights their versatility and ability to achieve both oncological safety and aesthetic outcomes [13,15,16,17,18,19,20,21]. First described by Hamdi et al. in 2006, these flaps are sourced from the chest wall or surrounding areas, such as the lateral thoracic or intercostal perforators, offering a reliable and versatile solution for volume replacement in BCS [11].

The minimal donor site morbidity, excellent aesthetic outcomes, and hidden scars of CWPFs make them an ideal choice for breast conservation, especially when the tumor-to-breast size ratio is high, and other techniques like therapeutic mammoplasty are unfeasible. CWPFs are particularly useful for defects in the inner breast quadrants, where alternatives like latissimus dorsi flaps may result in visible scarring. Moreover, autologous reconstruction with CWPFs avoids complications like capsular contracture and poor radiotherapy tolerance often seen with implant-based reconstructions [22]. In cases with small-to-moderate-sized breasts and large tumors, BCS can cause significant deformities or NAC deviation, but CWPFs provide a versatile solution that preserves symmetry.

In India, the adoption of CWPFs is crucial due to high mastectomy rates, advanced disease, and socio-economic challenges [23]. However, CWPFs remain underutilized due to limited awareness, surgeon training, and long-term data [24]. This study, the largest single-surgeon audit from Asia, aims to assess the feasibility and effectiveness of incorporating CWPFs into the standard oncoplastic armamentarium to improve breast cancer care. We present primary oncological outcomes, including recurrence rates and survival analysis. We also assessed secondary outcomes such as cosmetic results, post-op complications and PROM scores.

## 2. Methodology

### 2.1. Patient Selection

In the current retrospective observational study, patients were selected for CWPF surgery based on several criteria. The 200 patients included in the study were diagnosed with benign or malignant breast tumors, underwent thorough evaluation, and were found to be candidates for BCS with the need for volume replacement. These patients agreed to CWPF-based partial reconstruction in a shared decision-making process. Patients with an anticipated resection volume between 10–15% to 40–45% were included, encompassing those requiring central quadrant excisions and skin paddle excisions, where simple volume displacement would lead to asymmetry or shape distortion. Additional criteria for selection included multifocal/multicentric disease, large tumors, moderate tumors in patients with small breasts, post-NACT cases with a lukewarm response and large residual tumors, and cases where two flaps might be necessary for the defect. Once selected, all patients underwent a pre-operative donor site assessment and hand-held Doppler identification and marking of perforators.

### 2.2. Clinical Management

Oncological safety was rigorously maintained throughout the patient care pathway. Diagnosis relied on triple assessment. Confirmed cancer cases underwent surgery at a network hospital, with clinical staging guiding MDT-directed selection for NACT, NAHT, or adjuvant therapies per unit protocols and global guidelines. During surgery, ICG-guided lymph node clearance and flap vascularity assessments ensured precision.

Post-operative follow-ups over one month monitored early complications, while annual patient-reported outcome measures (PROMs) assessed quality of life. Cosmetic outcomes were independently evaluated by three surgeons.

### 2.3. Surgical Procedures

#### 2.3.1. Incision, Tumor Excision, and Oncological Clearance

**Figure 1:**
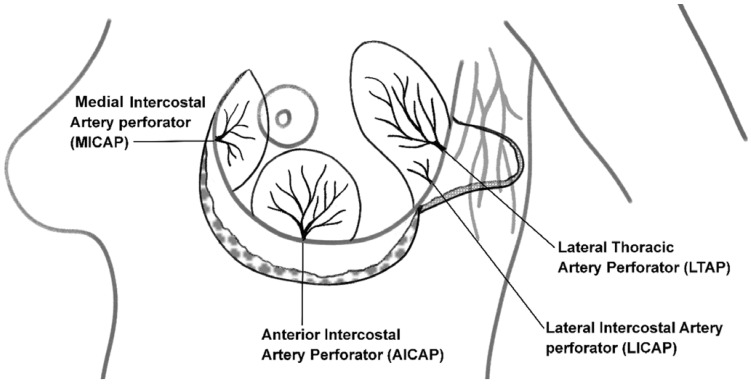
Schematic illustration of key perforators used in chest wall perforator flap breast reconstruction: The diagram highlights the anatomical location of perforator arteries utilized in chest wall perforator flap procedures.

Flap selection was based on tumor location and defect size, using single or combined perforators (LTAP, LICAP, MICAP, AICAP) to optimize volume and minimize scarring. Figure 1 is a schematic representation of the various perforators. Preoperative Doppler confirmed perforator presence, and patients were counseled on alternatives if needed.

On surgery day, perforators were re-confirmed and marked. SLNB was performed with ICG dye (AUROLAB, Madurai, India), and flaps were raised as planned. Tumor excision and axillary dissection adhered to oncological safety, with ICG assessing flap vascularity as well as perfusion assessment before final placement.

#### 2.3.2. Flap Selection

Depending on the tumor location, either LTAP, LICAP, MICAP, or AICAP were used as single perforators, or, in the case of larger defects, combinations to surcharge the flap with two perforators. Flaps are designed to provide volume or both volume and skin cover as required. LTAP and LICAP flaps are closed along the lateral mammary crease and under the bra line towards the axilla, while MICAP and AICAP flap donor sites are closed along the inframammary fold. The technique was tailored to provide volume replacement while minimizing and concealing scars. Representative cases for each perforator type are discussed to illustrate the technique.

Here, we specifically describe the LTAP perforator usage:

In our surgical methodology, we first identify the perforators and confirm the presence of the perforator. Preoperatively, we conduct Doppler assessment in the outpatient department (OPD) to ensure that the potential perforator is present. Once the presence of the perforator is confirmed in the OPD setting, we determine the appropriate flap to use for reconstruction and counsel the patient accordingly. For lesions in remote areas of the breast, such as the upper inner quadrant, where the presence and length of the Lateral thoracic artery Perforator (LTAP) is crucial, patients are informed about the possibility of not finding the LTAP and the alternative options available. These alternatives may include using a mini latissimus dorsi (LD) flap or thoracodorsal artery perforator (TDAP) flap, or repositioning the upper outer quadrant tissue into the defect and filling the gap with a lateral intercostal artery perforator (LICAP) flap. For tumors located in the upper outer quadrant or lower outer quadrant, a combination of upper outer quadrant tissue with LICAP can be utilized. In cases of large defects, multiple LICAP flaps can be designed and positioned as a mesentery. If the defect is in the lower outer quadrant, either a lower LICAP or anterior intercostal artery perforator (AICAP) flap may be used, depending on the specific location of the defect. These flap designs are finalized preoperatively in the OPD, and the patient is thoroughly informed about the surgical plan. On the day of surgery, we redefine the perforators in the recovery section to ensure precise execution of the planned reconstruction. We then mark out the axillary crease. Whether performing a LICAP or an LTAP, the upper incision is made obliquely to include the distal half of the crease in the LICAP. For an LTAP, the base is slightly higher on the lateral crease than for a LICAP, which is lower, with the flap extending obliquely towards the axillary crease.

With perforators marked, we use ICG dye for SLNB, injecting 0.05 mL at two subdermal points. The incision is made in the axillary lateral crease of the breast, extending obliquely upwards to the axillary crease, allowing access to the SLNB. Post-SLN dissection, we identify the LTAP, which lies around the base or median laterally and below the sentinal node, typically within the triangle formed by the subcutaneous nerve and the LTAP (lateral thoracic artery perforator). Using Doppler, we confirm the LTAP’s presence. If robust and supplying the flap, we proceed with tumor excision. Post-tumor excision, we re-enter the crease beside the tumor stringent rad-path analysis. We then identify and raise the LTAP flap from outside inwards, preserving vessels from the lateral side, including an accessory artery from the thoracodorsal muscle if present. Skeletonizing the flap, we maintain a tissue layer on the perforators. For axillary dissection, we perform it in reverse, detecting the LTAP peripherally and dividing the dissection into parts lateral and medial to the LTAP. Using appropriate equipment, we carefully dissect the LTAP, enabling thorough axillary dissection. Keeping the LICAP of the patient until the end, we retain the option of using LICAP. For inner quadrant tumors, we use the relay technique, filling gaps with LICAP. Post-flap raise, we inject ICG to assess vascularity. If a robust LICAP exists below, we clip it using a bulldog clamp to evaluate LTAP vascularity, exclusively. If the LTAP perforator is well functioning, we cut the mesentery, place the flap into the defect, and create a tunnel under the breast where the outer quadrant attaches to the skin.

#### 2.3.3. Pedicle Dissection

All flaps described in our study were harvested as true perforator flaps, with meticulous pedicle dissection up to their origin to ensure adequate vascularity. As noted in the surgical technique section, the LTAP and ICAP flaps were fully dissected up to their source vessels, ensuring robust perfusion, checked through ICG. The LTAP, for instance, was traced proximally to its origin at the axillary artery, and in select cases, mobilized into the muscle to enhance flap reach. Additionally, secondary pedicles were preserved when needed to maximize perfusion, particularly for larger reconstructions.

#### 2.3.4. Pre-Operative Markings

Tumor location, perforator location using Doppler, and flap incisions were marked preoperatively. The flap design aimed to incorporate scars into natural breast crease lines, minimizing visible scarring post-surgery.

#### 2.3.5. Tumor Localization

Tumor localization was guided by clinical examination and imaging. Impalpable lesions were localized pre-operatively using stereotactic wire placement with mammography or high-resolution ultrasound. Intra-operative ultrasonography (Fujifilm SonoSite Inc., Selangor, Malaysia) was used for sono-localizable tumors, while contrast-enhanced spectral mammography (CESM) (M/s. Bracco Suisse SA, Cadempino, Switzerland) aided in dense breast tissue evaluation. Post-NACT impalpable tumors were marked with clips placed pre- or mid-chemotherapy. The surgical sequence—tumor resection or axillary dissection first—was determined by tumor location, the need for SLNB or AD, and skin involvement.

#### 2.3.6. Axillary Management

Axillary management was guided by the need for sentinel lymph node biopsy (SLNB) or axillary lymph node dissection (ALND) based on tumor staging and clinical assessment. SLNB was performed for clinically node-negative patients using intraoperative ICG guidance. This involved injecting 0.05 mL of ICG dye at two subdermal points in the peripheral region to visualize the sentinel lymph nodes. For patients with positive SLNB or clinically positive axillary nodes, ALND was conducted. The axillary dissection was divided into two parts: lateral and medial to the LTAP, ensuring careful dissection around the LTAP to preserve its vascular supply to the flap. These guidelines ensured precise axillary management while maintaining optimal blood supply for the chest wall perforator flap (CWPF) techniques.

### 2.4. Post-Surgery Protocols

#### 2.4.1. Assessment of Post-Surgery Complications

Post-surgical outcomes were evaluated by breast oncoplastic surgeons and radiation oncologists. Complications were categorized according to the Clavien–Dindo classification system, with “major” complications requiring surgical intervention and “minor” complications managed conservatively. Additionally, the interval between the completion of surgery and the initiation of adjuvant therapy was monitored to identify any delays in the commencement of adjuvant treatments.

#### 2.4.2. Post-Surgery Marking of Tumor Bed for Radiotherapy and Adjuvant Radiation Therapy Methodology

The tumor bed is marked using Liga clips (Meril Endo Surgery Pvt. Ltd., Vapi, Gujarat, India) at the superior, inferior, medial, lateral, basal, and anterior margins to guide targeted radiotherapy. Based on our experience, the tumor margins remain within the original tumor volume, minimizing the risk of displacement to another quadrant.

RT aimed for a BED of 40 Gy in 15 fractions, with optional tumor bed boosts as needed. Treatment included the breast and, when required, the supraclavicular region, using F-P FiF IMRT or VMAT techniques. Immobilization employed Vac-Lok systems, and plans were created using Eclipse™ (F-P FiF IMRT) (Varian, CA, USA) or Monaco (VMAT) TPS (Elekta, Stockholm, Sweden). Dose delivery utilized the Elekta Medical System™ (Elekta, Stockholm, Sweden) with an 80-leaf MLCi. Plans ensured ≥95% PTV coverage, with permissible hotspots up to 110%. Tumor bed boosts followed standard protocols via electron portal or SIB techniques.

#### 2.4.3. Patient-Reported Outcome Measures

Patient-reported outcome measures (PROMs) were employed to gauge patient satisfaction and quality of life (QoL) following surgical procedures. The standardized BREAST-Q BCT module questionnaire was used to assess PROMs, with higher scores reflecting enhanced patient satisfaction and functionality.

### 2.5. Data Collection

Data were collected on patient demographics, tumor characteristics, medical history, clinical observations, pathological reports (including diagnostic biopsy and surgical histopathology with immunohistochemistry), details of neoadjuvant therapy, specifics of the surgical procedure, pre- and post-operative imaging, post-surgery complications, follow-up, and patient-reported outcome measures (PROMs). Clinical response to neoadjuvant chemotherapy (NACT) was categorized as pathological complete response (pCR) and residual disease (pRD).

### 2.6. Survival Analysis and Statistics

Data were retrospectively extracted from patient records, with recurrence identified through radiology, biopsy, or PET scans. Overall survival was assessed as all-cause mortality, calculated from surgery to death, based on information from relatives. Disease-free and overall survival percentages were calculated from survival tables based on median follow-up time.

## 3. Results

### 3.1. Overview of the Study Cohort

**Figure 2:**
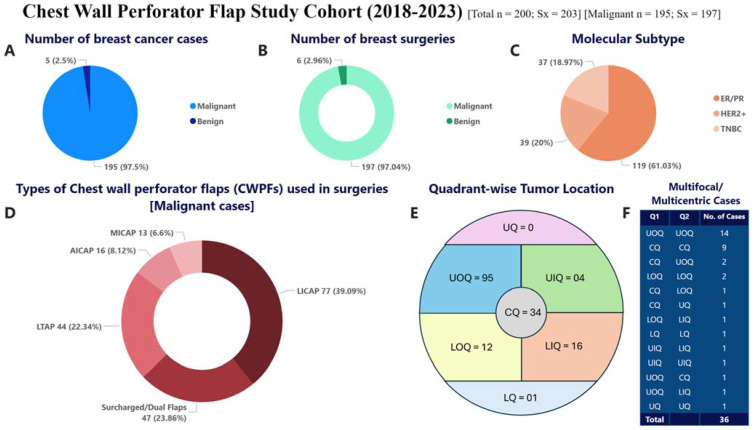
This figure highlights the clinicopathological distribution, types of CWPFs employed, and tumor location patterns, providing insights into surgical techniques and outcomes in breast conservation using CWPFs for our study cohort (2018–2023). (**A**) Number of breast cancer cases. (**B**) Number of breast surgeries. (**C**) Molecular subtype distribution. (**D**) Types of CWPFs used in malignant cases. (**E**) Quadrant-wise tumor location in Unifocal cases. (**F**) Quadrant-wise tumor location in multifocal/multicentric cases.

The demographic distribution of study participants is summarized in Figure 2 and Table 1. In the current study, 203 chest wall perforator flap procedures were performed for 200 breast disease patients from 2018 to 2023. Among the 200 patients, 192 presented with unilateral malignant disease, 2 patients had bilateral malignant disease, 5 patients were identified with unilateral benign disease, and 1 patient had one side malignant and one side benign disease. The median age at diagnosis of the patients included in the cohort was 52.5 years; the youngest was 31 years old and the oldest patient was 78 years old at the time of surgery. A proportion of the patients had comorbidities such as diabetes and hypertension, 41.5% (80/200), thus making them ineligible for mastectomy with immediate reconstruction.

Among 195 breast cancer patients (197 surgeries) (quadrant-wise tumor location is represented in Figure 2E), 56.3% (111/197) of tumors were observed in the upper quadrant. In our cohort, we observed 65% patients presenting at the cT2-T3 stage, and almost 50% of the cohort were clinically node positive. Table 2 summarizes the detailed description of the clinicopathological parameters of the breast cancer patients in the cohort.

Among the 197 surgeries for breast cancer, 150 underwent partial breast reconstruction using a single flap based on a single perforator, and 50 had it using combined flaps. All the six benign surgeries used a single flap based on a single perforator. Figure 2D represents the various types of chest wall perforators utilized as single or dual for the breast cancer patients. The subtype distribution for breast cancer patients in the cohort is represented in Figure 2C and Table 2. Figure 2E,F represent quadrant-wise tumor location in unifocal and multifocal/multicentric cases.

**Figure 3:**
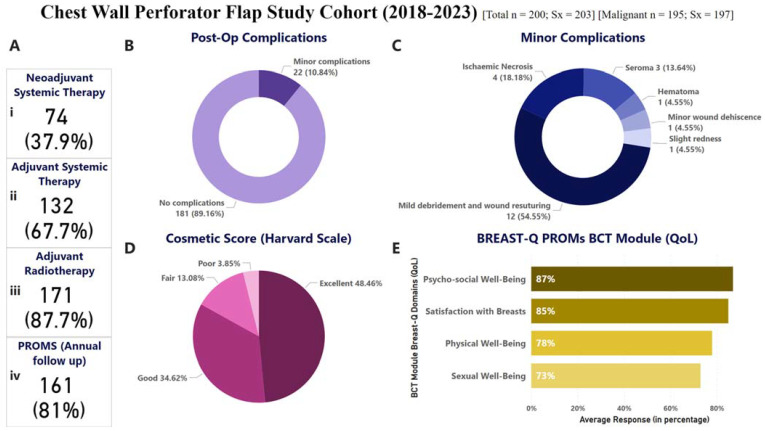
This figure presents outcomes and follow-up data from the chest wall perforator flap study cohort (2018–2023). (**A**) Neo and adjuvant therapy and follow-up statistics: (**Ai**) Neoadjuvant systemic therapy. (**Aii**) Adjuvant systemic therapy. (**Aiii**) Adjuvant radiotherapy. (**Aiv**) PROMs (Patient-Reported Outcome Measures). (**B**) Postoperative complications overview. (**C**) breakdown of minor complications. (**D**) Patient-Reported Outcome Measure (PROM) Scores. (**E**) Cosmetic outcomes (Harvard scale).

For the 195 breast cancer patients, median resection volume was 180 cc, and median pathological tumor size was 25 mm (range 1–65). Neo-adjuvant systemic therapy was administered to 74 (37.9%) patients, and 132 (67.7%) and 171 (87.7%) patients received adjuvant chemotherapy and adjuvant radiotherapy, respectively. In the cohort of 195 breast cancer patients, 40 patients fit the criteria for extreme oncoplasty. Figure 3 summarizes the clinical parameters of the breast cancer patients in the cohort.

### 3.2. Neoadjuvant Systemic Therapy (NAST—NACT/NAHT)

Of 195 breast cancer patients, 74 (37.9%) received NAST. Among the 74 patients who received NAST, pCR response was observed in 24.65% (19/74) patients. Pre-NAST mean clinical tumor size was 31 mm, while post-NAST mean pathological tumor size was 17.5 mm.

### 3.3. Surgical Outcomes

#### 3.3.1. Surgical Margins and Nodal Clearance

Clear margins were achieved in all the malignant patients. Of the 195 breast disease cases, axillary management was performed for 191 patients, of which only sentinel lymph node biopsy (SLNB) was done for 102 (53.4%), direct axillary lymph node dissection (ALND) for 41 (21.5%), and SLNB + ALND for 48 (25.1%).

#### 3.3.2. Post Operative Complications

In the current cohort of 200 cases undergoing PF surgeries, 22 cases (11%) developed post-operative complications, classified as per Clavien–Dindo Classification adapted for breast cancer [25]. A low rate of Grade I/II complications was observed immediately post-surgery, with all complications treated in outpatient settings. Figure 3B,C depict the post-operative complications observed in the current study.

### 3.4. Adjuvant Radiotherapy

Among the 195 breast cancer patients in our study cohort, 171 (88%) underwent adjuvant radiotherapy based on clinical indications. Of these, 112 patients experienced no adverse effects, while 59 developed mild-to-moderate (Grade I–II) radiation reactions. Notably, no cases of severe (Grade III) toxicity were observed. These findings indicate that the radiotherapy regimen was well-tolerated and safe across different CWPF procedures.

### 3.5. Survival Outcomes

For survival analysis, we are considering only 165 patients who have finished at least 1 year of follow-up post-surgery (2018–2022), with their treatment 12 months before this report. The median follow-up was 27 months for the survival analysis cohort.

**Figure 4:**
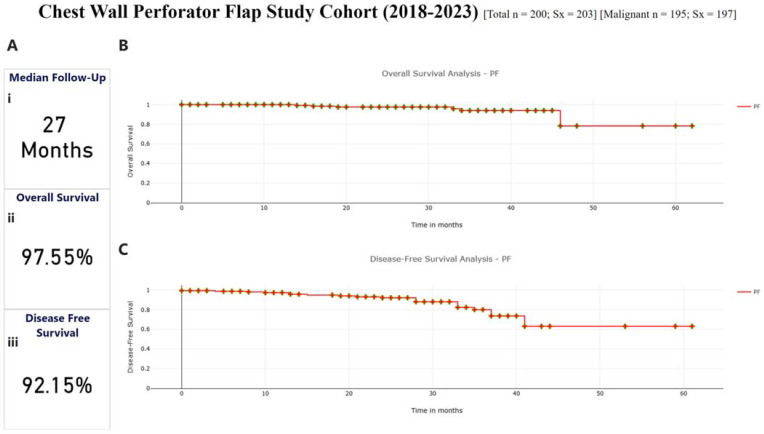
Kaplan–Meier plots for disease-free and overall survival (**Ai**) Median Follow-up For the Cohort (**Aii**) Overall Survival of 97.55% at Median Follow-up (**Aiii**) Disease-Free Survival of 92.15% at Median Followup. (**B**) Represents the overall survival graph for the cohort. The date of death due to any cause was taken as the event for overall survival with time calculated as the time period in months between the date of surgery and the date of death in months. Overall survival was 97.5% at median follow-up of 27 months. (**C**) Represents the Disease-Free Survival Curve for the cohort. Local and distant recurrences were marked as events for determining disease-free survival with time being calculated as the time period between surgery and the date of recurrence in months. Disease-free survival at 27 months median follow-up was 92.1%.

The overall recurrence rate was observed to be 12%, with 6.6% (11/165) local recurrence and 5.4% (9/165) distant recurrence, and overall mortality of 3.5% (6/165). Overall survival at median follow-up was 97.55%, and disease-free survival was 92.15%. Figure 4A–C depict the survival curves for the cohort.

### 3.6. Cosmetic Score Analysis

Out of 203 CWPF surgeries for 200 breast disease patients, cosmetic scores, assessed by surgeons within 3–6 months post-surgery, were good-to-excellent for 83% of the cases. Figure 3E shows the cosmetic scores as reported by the surgeons. Satisfaction with breasts in the Breast-Q PROM analysis also showed an average score of 85%.

### 3.7. Patient-Reported Outcome Measures (PROMs)

PROMs were assessed using BREAST-Q BCT module questionnaires after a minimum of 12 months post-surgery. Among the 200 patients in the study, 161 (81.0%) provided responses. The PROM data demonstrated high levels of patient satisfaction, as illustrated in Figure 3D.

## 4. Discussion

Numerous guidelines have been documented in the literature to determine the optimal approach for partial breast reconstruction, primarily based on the volume of excision, breast size, and degree of ptosis [26,27,28,29]. Lateral and anterior chest wall perforator flaps (CWPFs) have emerged as excellent options for partial reconstruction of small-to-moderate-sized breasts with limited ptosis, particularly for defects in the lateral and inferior quadrants. The evolution of Chest Wall Perforator Flaps (CWPFs) in breast reconstruction was initially described by Holmström and Lossing in 1986 for implant reconstruction [30]. This foundational work set the stage for subsequent advancements in CWPF techniques, particularly for the lateral thoracodorsal flap. Building on Holmström and Lossing’s work, Hamdi et al. (2004) adapted the lateral thoracodorsal flap for immediate partial breast reconstruction, demonstrating successful outcomes in the majority of cases [10]. By 2006, Hamdi et al. had extensively reported on AICAP flap reconstructions, presenting an initial series of 20 patients [11]. Their later study included 119 patients with a 4-year follow-up, comprising 93 cases of pedicled flap reconstructions using various techniques, all performed as one-stage reconstructions [31]. In 2012, Hamdi and Rasheed reviewed advancements in autologous breast reconstruction, highlighting that pedicled perforator flaps effectively address post-breast conserving therapy (BCT) defects while limiting donor site morbidity and preserving good aesthetic outcomes [32]. This study highlighted the clinical benefits of CWPFs, particularly their ability to avoid sacrificing the latissimus dorsi muscle, thus enhancing postoperative recovery and aesthetic results. McCulley et al. (2015) introduced LTAP flaps, reporting positive outcomes in 75 LICAP and LTAP cases [13]. Roy and Tenovici (2017) proposed a two-stage approach for high tumor-to-breast ratios, achieving a 10% complication rate in 20 patients [33]. Kim et al. (2018) demonstrated excellent outcomes in Korean women with small-to-moderate breast defects [34].

Recent studies support these findings > Soumian et al. (2020) reported minimal recurrence in a multicenter study of 112 patients but lacked cosmetic outcomes [15]. Carrasco-Lopez et al. conducted a parallel cadaveric and clinical study on AICAP flaps and concluded that AICAP flaps have consistent vascularization with good perforators, achieving satisfactory or excellent surgical and aesthetic outcomes [35]. Agrawal et al. (2020) confirmed these findings, reporting 95% excellent or good cosmetic results and high patient satisfaction in 40 cases, reinforcing CWPFs as a valuable tool in oncoplastic surgery [18]. Kabeer et al. (2022) analyzed 152 CWPF reconstructions, showing reduced mastectomy rates and lower morbidity [23]. Gupta Roy et al. (2021) followed 105 patients for 54 months, reporting high satisfaction [36], while Orabi et al. (2022) observed 90% satisfaction in 26 cases [37]. Two recent studies further validate these results: Agarwal et al. (2024) analyzed 150 cases in India, and Karakatsanis et al. conducted a multicenter study with 603 patients, demonstrating minimal morbidity and excellent oncological and aesthetic outcomes for breast conservation surgery with CWPFs [17,19]. Collectively, these studies affirm CWPFs as a cornerstone in partial breast reconstruction.

We have previously reported the use of oncoplasty in managing large breasts with ptosis as well as use of oncoplasty in extreme cases. To our knowledge, the current study is the largest cohort from India discussing management of breast cancer with Level 3 oncoplastic techniques [38], thus adding to our toolkit of oncoplastic techniques for managing various scenarios in breast cancer in the Indian context.

Our study represents a retrospective audit of 200 patients who underwent CWPF based partial breast reconstructions (2018–2023), and to the best of our knowledge, it is the largest reported series to date. It highlights the efficacy of CWPFs as a favorable option for partial breast reconstruction, with satisfactory patient-reported aesthetic outcomes and minimal morbidity, performed by a single oncoplastic breast surgeon. Intraoperative techniques, including acoustic Doppler confirmation and potentially ICG dye utilization, contribute to minimizing complications and enhancing procedural precision. A noteworthy proportion of patients presented with histologically larger tumors, potentially obviating the necessity for mastectomy via CWPF-based reconstructions. Margin revision, necessitated in a minority of cases (0.5%), incurred minimal additional morbidity, with only one patient requiring complete mastectomy due to persistent positive margins. In our experience, we found CWPF-based partial reconstructions to offer remarkable versatility in breast conservation surgery, for small-to-moderate-sized breasts requiring extreme excisions (large tumors > 5 cm), smaller multifocal/multicentric tumors, or extensive ductal carcinoma in situ (DCIS) (Case 1 and 5—Supplementary Figures S1 and S5, Supplementary Videos S1 and S5, Supplementary Data S1). CWPFs have been invaluable during intraoperative surprises, where unexpected tumor extent necessitated larger resections than initially planned, either de novo or following NACT. In post-NAST scenarios, CWPFs have significantly increased BCS rates by facilitating conservation in cases of suboptimal response, including large residual tumors, honeycomb-like changes, or extensive calcifications post-NAST (Case 2—Supplementary Figure S2, Supplementary Data S1, Supplementary Video S2). Additionally, they have been instrumental in skin replacement and in managing tumors located in difficult quadrants, such as the upper inner, inferior, central, and axillary regions, where their adaptability to complex anatomy has ensured superior outcomes (Case 2—Supplementary Figure S2, Supplementary Data S1). Beyond primary reconstruction, CWPFs have also been employed for corrective surgeries, effectively addressing poor excision biopsy scars and salvaging necrotic autologous flaps in whole breast reconstruction. Their transformative potential extends further, establishing CWPFs as a reliable reconstructive tool in diverse and challenging scenarios, as demonstrated through several representative cases in our practice. Its application also extends to managing challenging quadrants, such as the upper inner quadrant, inner quadrant, central quadrant, and axillary tumors (Case 3 and 4—Supplementary Figures S3 and S4 and Supplementary Video S3 and S4, Supplementary Data S1), and offers a reliable reconstructive option for poor excision biopsy scars, providing effective skin replacement and addressing aesthetic concerns associated with extensive tissue removal. The technique ensures minimal scarring on the breast, with scars discreetly hidden on the lateral chest wall or beneath the breast in IMF, maintaining the aesthetic appeal. CWPF also enables surgeons to achieve symmetry in breast size and volume, providing balanced and natural outcomes. In addition to their role in partial breast reconstruction, CWPFs have demonstrated remarkable versatility in our practice. In our experience, they can also be employed as an alternative to acellular dermal matrices (ADMs), which are not readily available in India, for autologous reconstruction and in the correction of necrotic flaps. Additionally, CWPFs have also demonstrated exceptional value in corrective surgeries, particularly in salvaging necrotic autologous flaps used for whole breast reconstruction and restoring tissue integrity effectively. Through representative cases from our practice, we highlight the versatility of CWPFs, demonstrating their application across different perforators and addressing some of the key clinical situations.

### Surgeons’ Recommendations for Young Surgeons on CWPF Surgical Algorithm

Preoperative Planning:
  - Precisely identify the LTAP preoperatively to ensure its course to the lateral fold.
  - Use high-resolution imaging techniques (ultrasound, contrast mammography) to map out the tumor and perforators.
Marking and Incision:
  - Mark the axillary crease carefully.
  - For LTAP, make a small incision that includes the sentinel node biopsy site and allows dissection up to the perforator’s origin.
Flap Dissection:
  - Begin flap dissection from the lateral to medial side for clear visualization and controlled handling of the perforator.
  - Dissect the LTAP perforator up to its origin in the axillary artery to increase flap mobility and reduce traction.
  - Mobilize the perforator within the muscle if additional reach is needed.
ICG Utilization:
  - If the facility is available, use Indo cyanine dye for SLN mapping.
  - Inject ICG dye subdermally to identify SLN and assess vascularity pre- and post-dissection.
  - Confirm perfusion of the LTAP flap to avoid under-perfused areas.
Flap Placement:
  - Tunnel the flap through the subcutaneous space to the defect site, ensuring adequate reach and minimal traction on the breast.
  - Avoid fixing the flap tip directly to breast parenchyma to prevent asymmetry. Secure the flap to the chest wall using retaining sutures on superior and inferior borders.
Margins and Tumor Resection:
  - Maintain wide margins guided by intraoperative imaging and frozen section analysis to ensure complete tumor excision.
Axillary Dissection:
  - When required, perform axillary dissection in two stages: lateral and medial to the LTAP, ensuring meticulous preservation of the perforator.
Supercharging:
  - For larger defects, consider supercharging the LTAP with an additional LICAP to enhance vascularity.
Validation and Documentation:
  - Validate margins using frozen sections, and follow up with paraffin section confirmation.
  - Rely on specimen imaging and pathology to confirm adequacy of resection and flap coverage.
Patient-Specific Adjustments:
  - Adapt flap size based on tumor location (e.g., smaller flaps for medial quadrant tumors).
  - Ensure clear communication about surgical plans and outcomes to manage patient expectations effectively.

While the current study provides valuable insights into the use of CWPFs in breast-conserving surgery, we acknowledge certain limitations in this study.

Retrospective Design—As a retrospective study, data collection is subject to inherent biases, including selection and information bias.

Single-Center Study—The findings are based on a single institution’s experience, which may limit generalizability to other centers with different patient populations, surgical expertise, and healthcare infrastructure.

Lack of a Control Group—The absence of a direct comparison with other reconstructive/OBS techniques or standard breast conservation surgery without CWPFs limits the ability to determine relative advantages and long-term superiority.

Short to Intermediate Follow-Up—While oncological and patient-reported outcomes are reported, longer follow-up is needed to assess late recurrences, flap durability, and long-term cosmetic outcomes.

Potential Selection Bias—Patients were selected based on clinical suitability for CWPFs, which may introduce selection bias and limit the applicability of findings to all breast cancer patients undergoing breast conservation.

Our results advocate for the consideration of CWPF-based reconstructions as a viable option in breast surgery, particularly in cases where conventional approaches may necessitate more extensive resections. Further prospective studies are warranted to validate these results and refine surgical protocols.

## Figures and Tables

**Table 1:** Demographic features of the cohort.

Feature	Class	N = (200)
Age (years)	Median (Range)	52.5 (31–78)
	<40	24
	41–60	127
	>60	49
Comorbidities	Yes	80
	No	119
	NA	1
Size of Breast	S	28
	M	150
	L	18
	NA	4
Ptosis	No Ptosis	34
	Grade I Mild	68
	Grade II Moderate	46
	Grade III Severe	48
	NA	4
Malignant/Benign	Malignant	195
	Benign	5

**Table 2:** Clinicopathological features of the breast cancer patients in the cohort.

Feature	Class	Total N = 195 Sx = 197 Malignant	
Molecular Subtype	ER/PR	119	61.03%
	HER2	39	20%
	TNBC	37	18.97%
Focality	Unifocal	155	81%
	Multifocal/Multicentric	37	19%
In Unifocal Clinical Tumor Size (cT)	cT1	51	25%
	cT2	86	43%
	cT3	11	5%
	NA	5	-
In Multifocal Clinical Tumor Size (cT)	cT1	11	5%
	cT2	22	10%
	cT3	3	1.5%
DCIS	Tis	8	4%
Tumor Grade	I	8	4%
	II	114	58%
	III	55	28%
	NA	20	-
Type of Tumor (Biopsy)	IDC	156	79.2%
	IDC + DCIS	25	12.8%
	ILC	4	2%
	ILC + LCIS	1	0.5%
	DCIS	9	4.5%
	Others	2	1%
Quadrant (unifocal) (n = 160)	UOQ	95	59%
	CQ	34	21%
	LIQ	16	10%
	LOQ	12	7.5
	LQ	1	0.6%
	UIQ	4	2%
	UQ	0	-
Clinical Tumor Stage (Underwent Upfront Surgery) 121/195 (62%) patients underwent upfront surgery	Stage 0	0	-
	Stage IA	25	20%
	Stage IB	0	-
	Stage IIA	45	37%
	Stage IIB	25	20%
	Stage IIIA	12	10%
	Stage IIIB	0	-
	Stage IIIC	2	1.6%
	NA	6	-
Clinical Node Positivity (Upfront Surgery)	42 of 121 (34.71%) were node positive		
Clinical Tumor Stage (Given NAST) 74/195 (37%) patients received NAST	Stage 0	1	1.3%
	Stage IA	5	6%
	Stage IB	0	-
	Stage IIA	18	24.3%
	Stage IIB	19	25.6%
	Stage IIIA	29	39.1%
	Stage IIIB	0	-
	Stage IIIC	2	2.7%
	NA	0	-
Clinical Node Positivity (NAST)	55 of 74 (74.32%) were node positive		
Pathological Tumor Stage (Upfront Surgery)	Stage 0	9	
	Stage IA	20	
	Stage IB	0	
	Stage IIA	43	
	Stage IIB	29	
	Stage IIIA	8	
	Stage IIIB	1	
	Stage IIIC	4	
	NA	7	
Pathological Tumor Stage (Post-NAST Surgery)	Stage 0 (pCR)	17	
	Stage IA	14	
	Stage IB	0	
	Stage IIA	20	
	Stage IIB	10	
	Stage IIIA	6	
	Stage IIIB	0	
	Stage IIIC	5	
	NA	2	
Post-Op Complications	No Complications	178	
	Grade I Complications	17	
	Grade II Complications	5	

## Data Availability

The data presented in this study are available on request from the corresponding author due to ethical and privacy concerns.

## Supplementary Materials

The following supporting information can be downloaded at: https://zenodo.org/records/14769470 (accessed on 18 February 2025).

## Abbreviations

AD	Axillary Dissection
ADM	Acellular Dermal Matrices
AICAP	Anterior Intercostal Artery Perforator Flap
ALND	Axillary Lymph Node Dissection
BC	Breast Cancer
BCS	Breast Conservation Surgery
BCT	Breast Conservation Therapy
CESM	Contrast Enhanced Spectral Mammography
CWPF	Chest Wall Perforator Flaps
DCIS	Ductal Carcinoma In Situ
ICG	Indocyanine Green
IDC	Intraductal Carcinoma
IMRT	Intensity-Modulated Radiation Therapy
KM	Kaplan–Meier
LABC	Locally Advanced Breast Cancer
LD muscle	Latissimus Dorsi muscle
LICAP	Lateral Intercostal Artery Perforator Flap
LTAP	Lateral Thoracic Artery Perforator Flap
MDT	Multi-disciplinary Team
MICAP	Medial Intercostal Artery Perforator Flap
NAC	Nipple Areolar Complex
NACT	Neo-Adjuvant Chemotherapy
NAHT	Neo-adjuvant Hormone Therapy
NAST	Neo-adjuvant Systemic Therapy
OBS	Oncoplastic Breast Surgery
pCR	Pathological Complete Response
PET	Positron Emission Tomography
pRD	Pathological Residual Disease
PROMs	Patient Reported Outcome Measures
QoL	Quality of Life
RT	Radiation Therapy
SIB	Simultaneous Integrated Boost
SLNB	Sentinel Lymph Node Biopsy
TPS	Treatment Planning Software
TRM	Therapeutic Reduction Mammoplasty
UOQ	Upper Outer Quadrant
VMRT	Volumetric Modulated Arc Therapy
